# Specificities of Scanning Electron Microscopy and Histological Methods in Assessing Cell-Engineered Construct Effectiveness for the Recovery of Hyaline Cartilage

**DOI:** 10.3390/mps4040077

**Published:** 2021-10-27

**Authors:** Mikhail S. Bozhokin, Svetlana A. Bozhkova, Aleksandr A. Rubel, Julia V. Sopova, Yulia A. Nashchekina, Natalya B. Bildyug, Mikhail G. Khotin

**Affiliations:** 1 Vreden National Medical Research Center of Traumatology and Orthopedics, Academica Baykova Str., 8, 195427 St. Petersburg, Russia; clinpharm-rniito@yandex.ru; 2 Institute of Cytology, Russian Academy of Sciences, Tikhoretsky Ave. 4, 194064 St. Petersburg, Russia; y.sopova@spbu.ru (J.V.S.); nashchekina.yu@mail.ru (Y.A.N.); nbildyug@gmail.com (N.B.B.); Khotin@incras.ru (M.G.K.); 3 Laboratory of Amyloid Biology, St. Petersburg State University, Universitetskaja Emb., 7/9, 199034 St. Petersburg, Russia; a.rubel@spbu.ru; 4 Department of Genetics and Biotechnology, St. Petersburg State University, Universitetskaja Emb., 7/9, 199034 St. Petersburg, Russia; 5 Research Center for Genetics and Life Sciences, Sirius University of Science and Technology, Olympiiskii ave., 1, 354340 Sochi, Russia; 6 Center of Transgenesis and Genome Editing, St. Petersburg State University, Universitetskaja Emb., 7/9, 199034 St. Petersburg, Russia; 7 Vavilov Institute of General Genetics, St. Petersburg Branch, Universitetskaja Emb., 7/9, 199034 St. Petersburg, Russia

**Keywords:** SEM, histology, cellular engineered constructs, tissue engineering, hyaline cartilage

## Abstract

Damage to the hyaline layer of the articular surface is an urgent problem for millions of people around the world. At present, a large number of experimental methods are being developed to address this problem, including the transplantation of a cell-engineered construct (CEC) composed of a biodegradable scaffold with a premixed cell culture into the damaged area of the articular surface. However, current methods for analyzing the effectiveness of such CECs have significant limitations. This study aimed to compare the SEM technique, classical histology, and cryosectioning for the analysis of CECs transplanted to hyaline cartilage.

## 1. Introduction

Damage to the hyaline layer of the cartilage articular surface is an urgent problem for millions of people around the world [1]. Due to its avascular structure and the high exposure of cartilage to mechanical load, its regenerative potential is extremely low [2], as shown in many studies [3,4,5], including our own work [1,6,7]. A large number of techniques for cartilage restoration are currently available for surgeons. However, they are still not sufficiently effective [8]. To restore the damaged articular surface, cell engineering methods provide a promising approach and involve the preparation of a biodegradable and safe scaffold colonized with a culture of stimulated cells and subsequent transplantation into the damage area [9,10,11]. Scaffold protects the cells from significant mechanical stress on the hyaline cartilage surface and promotes cell proliferation in 3D. Stimulation of cell cultures (MSC or chondrocytes) may be undertaken in different ways, including the use of Tgfβ3 (transforming growth factor β3), the key cytokine for chondrogenesis [12]. Proliferating cells synthesize a large amount of the hyaline cartilage extracellular matrix. The use of such cell-engineered constructs (CECs) is justified in traumatology and orthopedics, since it makes it possible to replace the damaged and avascular tissue area with an autologous graft similar to the native tissue in terms of both physical/mechanical properties and biological parameters.

## 2. Materials and Methods

### 2.1. Cell Cultures

Multipotent mesenchymal stromal cells (MSCs) were isolated from the femur bones of adult rats (6 months old). Briefly, rats were euthanized with i.p. administration of thiopental sodium. The femur bone was extracted from the knee and hip joints without damaging the bone itself. Next, the bone was washed with antibiotic streptomycin-penicillin solution (50,000 units/mL of penicillin and 25,000 µg/mL of streptomycin; Biolot, Saint-Petersburg, Russia). The upper and bottom bone parts were cut off, and the MSCs cells were washed out with growth media (DMEM; Biolot, Saint-Petersburg, Russia), 15% FBS (Biolot, Saint-Petersburg, Russia), and the antibiotic streptomycin-penicillin solution. MSCs were plated on adhesive culture plastic (Sarstedt, Numbrecht, Germany) and cultured for two weeks (three passages). The culture medium was changed every three days. The cells were grown to 80% confluency and passaged. MSC status was confirmed by flow cytometry using monoclonal anti-CD45 (#554878, BD Biosciences, San Diego, CA, USA) and anti-CD90 (#554897, BD Biosciences, San Diego, CA, USA) antibodies conjugated with phycoerythrin (PE) and fluorescein isothiocyanate (FITC) fluorochromes, respectively, on a BD FACS Aria III flow cytometer-cell sorter (BD Biosciences Div. 7, San Diego, CA, USA) using FACS Diva 7 software (Denovo Software, Los Angeles, CA, USA). Cultured cells were stimulated using 10 ng/mL Tgfβ3 cytokine (Sigma, St. Louis, MO, USA) at each medium change for one week. Additional components of the stimulation mixture, including L-prolin (50 μg/mL; Sigma-Aldrich, St. Louis, MO, USA), ascorbic acid (50 μg/mL; Sigma-Aldrich, St. Louis, MO, USA), and dexamethasone (100 nmol/mL; Sigma-Aldrich, St. Louis, MO, USA), were mixed immediately before each addition to the medium.

### 2.2. Scaffold Preparation

The scaffold was produced by freeze-drying. Poly-L-lactide (Mn 20,000, Sigma, St. Louis, MO, USA) was dissolved in a 95:5 1,4-dioxane (Sigma, St. Louis, MO, USA)/distilled water solution at 60 °C. Once dissolved, the polymer solution was transferred into the mold and frozen at −20 °C. The samples were lyophilized until complete removal of the solvent had been achieved. We prepared the mold ourselves from polyethylene terephthalate (Figure S1). With the help of a special machine, the inner and outer surfaces of the mold were polished. The outer height of the mold was 7 mm and the inner height was 5 mm.

### 2.3. Preparation of a Cell-Engineered Construct

A specially device developed in-house was used to combine the biodegradable scaffold and cell culture (Figure S2) [13]. Before use, the device was disinfected by washing with 70% ethanol and autoclaved at 130 °C for 2 h. Next, a blank scaffold was inserted and a growth medium with 0.5 × 10^6^ cells/mL MSCs was forced onto it using a pump with the filtered medium volume of 5 mL. After that, the apparatus was sealed, and high-purity nitrogen with an overpressure of 0.1 MPa was supplied through a solenoid valve with an external control, with a double filtration of the cell culture. The number of cells was measured using a Countess^®^ device (Thermofisher, Carlsbad, CA, USA) before colonization, after the first stage, and after the second stage of filtration. The CEC was stimulated using 10 ng/mL Tgfβ3 cytokine (Sigma, St. Louis, MO, USA) at each medium change for 2 weeks before the transplantation. Additional components of the stimulation mixture, including L-prolin (50 μg/mL; Sigma-Aldrich, St. Louis, MO, USA), ascorbic acid (50 μg/mL; Sigma-Aldrich, St. Louis, MO, USA), and dexamethasone (100 nmol/mL; Sigma-Aldrich, St. Louis, MO, USA), were mixed immediately before each addition to the medium.

### 2.4. Animal Studies

Animal studies were conducted on adult Wistar rats (6 months old) in accordance with current ethical standards. The rats (experimental unit = single rat) were obtained from a specialized nursery for experimental and laboratory animals, “Rappolovo” (Federal State Unitary Enterprise “Nursery of laboratory animals Rappolovo”, Russia, Leningrad region, Vsevolozhsky district, Rappolovo village). Animals were kept in quarantine for 21 days after arrival and then were maintained under standard conditions. The animals were 6–month-old female rats weighing 200–210 g. Animals had no genetic or other modifications. The animals were maintained under appropriate conditions: natural humidity and a temperature of 18 °C, with artificial lighting for 18 h a day and forced ventilation. For in vivo experiments, all animals were randomized into two groups (control (C) and experimental (E), n = 12 animals per group, 24 animals in total), and all animals were analyzed (there were no exclusion criteria in our experiments). In all animal groups (both control and experimental), surface defects consisting of loaded zones of the femur hyaline cartilages were formed using a bur. For this, the joint capsule of an experimental animal anesthetized with a mixture of ketamine (50 mg/mL, 0.5 mL) and sibazone (5 mg/mL, 0.5 mL) was opened using an external parapatellar method, and a standard defect was formed using a dental bur and an original device to create standardized lesions [14]. The diameter of defects was 1000 μm and their depth was 500 μm. In the experimental group, transplantation of CECs, stimulated with the Tgfβ3 cytokine, was additionally performed. In the control group, there was no transplantation of CECs in the defect area. All animal groups were withdrawn from the experiment at day 90 by injecting an increased dose of sodium thiopental, and the knee joint samples were analyzed with scanning electron microscopy (SEM) and histological methods (HMs).

### 2.5. Scanning Electron Microscopy

SEM was performed on a Jeol JSM6390LA system (Jeol, Tokyo, Japan) in cooperation with the Komarov Botanical Institute of the Russian Academy of Sciences (St. Petersburg, Russia). For this, 5–8-mm-thick cube-shaped tissue samples were isolated. Then, tissue samples were dehydrated in ascending alcohol concentrations of 50%, 70%, and 96%. Each stage lasted 24 h. The last dehydration was repeated once, since the SEM technique requires complete dehydration. After that, the samples were finally dried under vacuum. Careful and gradual dehydration of the preparations was a key step. Next, a 5-nm-layer of palladium and gold was sprayed using vacuum deposition onto the preparation. An electron beam was directed toward the SEM preparations and the surface, featuring either regenerative or degenerative changes in the hyaline cartilage or CECs, was visualized (surface imaging). The changes in hyaline cartilage were analyzed according to the International Society for Cartilage Research (ICRS) scale [15]. When evaluating the results obtained by histological methods and scanning electron microscopy, all researchers were aware of all experimental stages.

### 2.6. Classical Histological Studies

For classical histological studies, a joint fragment was placed in the Trilon B decalcifying solution for five days (Biolot, Saint-Petersburg, Russia) with a preliminary fixation in formalin solution for three days. After the decalcification procedure and medium change (CECs were not decalcified), the preparations were placed into disposable plastic cassettes for histological studies in standardized orientation, with mandatory control of the defect area for the standard orientation of subsequent sections. To analyze the cell proliferation within the CECs, cells were stained with eosin dye (Bio-Vitrum, Saint-Petersburg, Russia), providing a pink color in contrast to a colorless paraffin block. The sample was embedded into paraffin and sections of 10 μm were prepared using a Leica sled microtome (Leica, Wetzlar, Germany). Staining with hematoxylin and eosin (Bio-Vitrum, Saint-Petersburg, Russia), as well as Alcian blue (Bio-Vitrum, Saint-Petersburg, Russia), was performed to identify glycosaminoglycans in accordance with the manufacturer’s protocols. Hyaline cartilage changes were assessed using a modified O’Driscoll scale [16]. However, when performing histological preparations of blocks obtained from experimental animals, there were difficulties with cutting out the region of interest due to the small size of the histological preparation.

### 2.7. Cryosectioning

During the production of cryosections, the preparations were embedded in FSC22 BLUE compound (Leica, Wetzlar, Germany) for encapsulation and contrast visualization of the object. The blue color of the compound provided additional contrast to the light-colored biodegradable carrier. Then, flash-freezing in liquid nitrogen was performed for 1 min. Sections were prepared on a Leica CM1850UV cryomicrotome (Leica, Wetzlar, Germany). Section thickness varied between 5 and 20 microns. Slides with an additional polylysine layer were exclusively used to improve the adhesion of the cryosection. An additional polylysine layer for adhesion was provided by applying a polylysine solution (0.1% polylysine aqueous solution; Sigma, St. Louis, MO, USA) and drying for 24 h at 37 °C. Then the cryosection was placed on the slide, stained (hematoxylin and eosin, Alcian blue), and dried in a strictly horizontal position to prevent it from peeling off the slide.

### 2.8. Confocal Microscopy

On the seventh day of CEC culturing, cryosections were made and stained with a fluorescent DAPI dye (nuclear staining). Confocal microscopy was performed on a Leica TCSSP5 (Leica, Wetzlar, Germany). In this work, we did not use statistical research methods due to the small number of samples analyzed at each time point for each method. However, the evaluation of results (and scoring) was carried out by six researchers independently, and the results were averaged only after that.

## 3. Results

**Figure 1:**
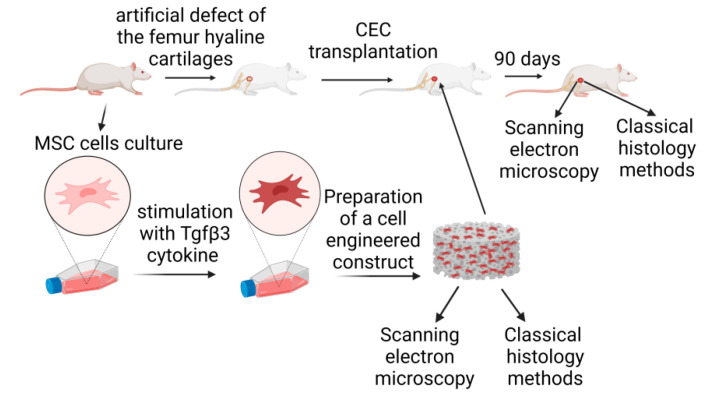
The assessment of cell-engineered construct effectiveness for the regeneration of hyaline cartilage.

MSCs were isolated from the femur bone marrow of adult rats and cultured for three passages. MSC identity was confirmed by the expression of the CD90 marker with the absence of the CD45 marker using flow cytometry. In cell culture stimulated with Tgfβ3 cytokine, the formation of cell aggregates could already be observed on day 7, indicating the onset of chondrogenic differentiation. Successful isolation of cell culture allowed the initiation of the CEC colonization using the device developed in-house. The overall scheme of the experiment is presented in Figure 1.

### 3.1. SEM Analysis of Scaffold and CEC Surface

**Figure 2:**
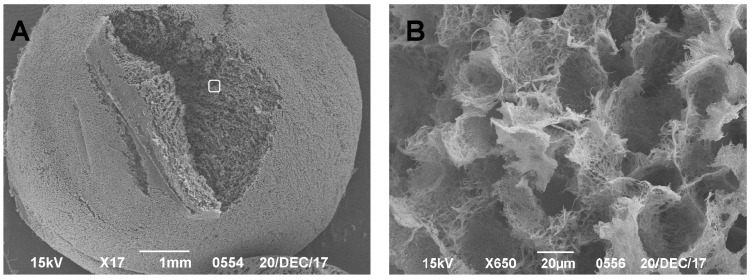
(**A**) SEM images of a polylactide matrix without cell elements; scale bar of 1 mm is shown. (**B**) Higher magnification of the boxed area in (**A**).

**Figure 3:**
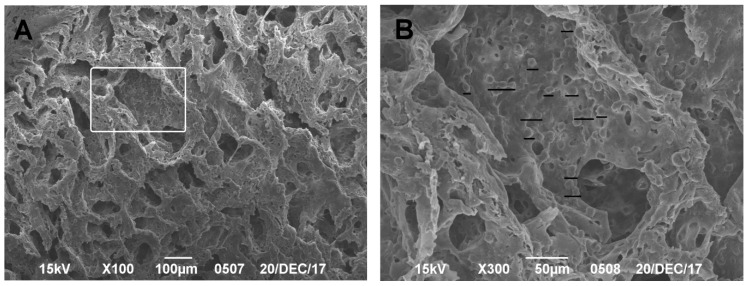
(**A**) SEM image of the CEC with cells on the seventh day of observation; scale bar of 100 µm is shown. (**B**) Higher magnification of the boxed area in (**A**); cells covering the scaffold are underlined.

**Figure 4:**
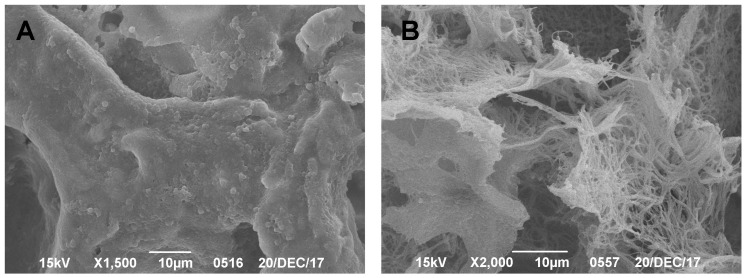
SEM image of the CEC with cells on the 14th day of observation; scale bar of 10 µm is shown. (**A**) CEC. (**B**) Polylactide matrix in a native (not cell-colonized) state.

During the production of SEM preparations, no destructive processes were observed in the samples tested. This technique made it possible to visualize the surface structure of the native matrix at different magnifications and to analyze the porosity and structural features of the polylactide carrier: pore connectivity, size, and location. In contrast to the non-colonized samples (Figure 2), the colonized matrix samples (Figure 3 and Figure 4) showed the distributions of cells on the surface and in the near-surface layers of the scaffold, which confirmed the effectiveness of the device developed in-house for dynamic cell colonization.

### 3.2. Histological Methods

The use of an original device [14] for the dynamic colonization of a biodegradable carrier with cell culture enabled the efficient distribution of the experimental cells throughout the entire volume of a matrix composed of PLA (polylactide)-based polymer. Cells inside the polylactide scaffold were visualized histologically; however, during the histological processing, partial biodegradation of the polymer carrier was observed as a result of xylene exposure.

**Figure 5:**
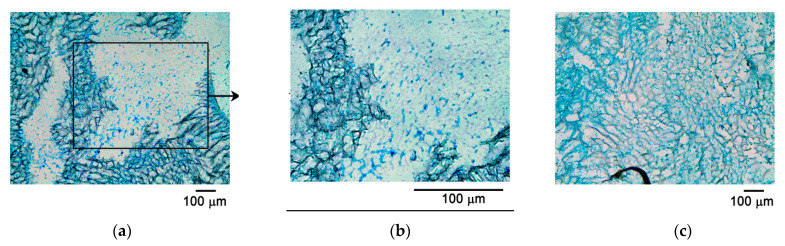
Histological cryopreparation of CEC with cells at (**a**,**b**) day 7 and (**c**) day 14; Alcian blue staining.

Another method analyzed was the method of producing cryosections without standard histological processing. During the preparation of the cryomicrosection, there were no signs of sample degradation, in contrast to the classical histology method. Microscopy of the obtained preparations also confirmed the colonization of the CEC with MSC culture (Figure 5) throughout the matrix. Alcian blue staining demonstrated the presence of synthesized hyaline-cartilage extracellular matrix in the analyzed CECs (Figure 5b). In addition, the preservation of the carrier structure made it possible to confirm the proliferation of cells within the CEC and to establish the dynamics of cell proliferation, whereby cells occupied the entire space inside the scaffold by the 14th day of culturing (Figure 5a,c).

### 3.3. Confocal Microscopy

**Figure 6:**
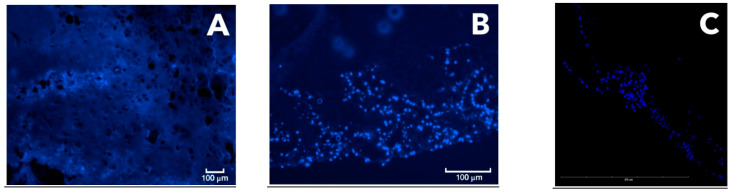
(**A**,**B**) Fluorescent microscopy of the CEC cryosection on day 7 and (**C**) confocal microscopy of CEC samples on day 7; Dapi staining.

Analysis with confocal and fluorescent microscopy after preparation of cryosections also confirmed the viability and proliferation of the cell culture inside the biodegradable carrier on the seventh day of CEC culturing. Staining revealed the uneven colonization of the CEC with MSC culture. For example, higher densities were observed in the membrane center and along the edges (Figure 6A,B). Some of the sections were examined using a confocal microscope and the corresponding images were obtained. Using this technique, it was possible to separate a rather intensive background fluorescence of the polylactide carrier from the signal of the fluorescent label, thus obtaining a more informative image (Figure 6C). This technique made it possible to evaluate the CEC’s 3D structure through sequential analyses of different layers, thus producing a 3D image.

### 3.4. SEM Results of CEC Transplantation Can Be Scored Using the Established ICRS System

**Figure 7:**
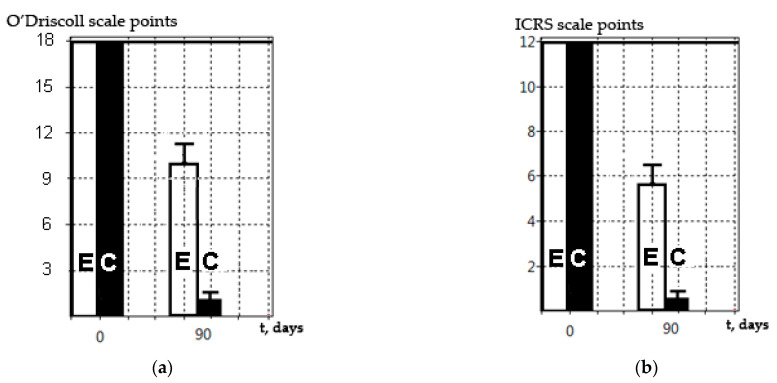
Assessment of the Tgfβ3-modified CEC application according to the O’Driscoll scale with modifications for histological methods (**a**) and the ICRS scale for SEM methods (**b**). Solid line is the result for the intact hyaline cartilage. E—experimental group with CEC transplantation. C—control group, only defect.

In all experimental animals, perifocal reactions were observed in the area of the simulated defect. In the control group (only the defect without CEC transplantation), significant degenerative damage to both the surface layer of the hyaline cartilage and the underlying subchondral layer was observed at day 90 using histological and SEM methods. In the experimental group with Tgfβ3-stimulated CEC transplanted into the defect area, the damage was repaired by the regenerated tissue (see Figures S3 and S4). SEM methods revealed that its surface structure corresponded to the intact hyaline cartilage. HMs with Alcian blue staining showed a significant amount of glycosaminoglycans in the regenerated area, as well as hyaline-like tissue on the surface. A comparison of the data obtained by SEM and HMs on the ICRS and O’Driscoll scales showed similar correlating results (Figure 7).

## 4. Discussion

The principles of tissue engineering were formulated by Langer in 1993 [17]. The restoration of defects in different organs and tissues using CECs is now widely performed; in particular, for the replacement of defects in the hyaline layer of articular cartilage [18]. To provide a functional CEC, a safe and biodegradable scaffold for the cell culture should be used [16]. The data from this article are consistent with literature data, as well as our own previous studies [19]. Currently, different methods are used for in vitro as well as in vivo assessment of CECs; however, all of them have significant limitations and application specificities.

Here, we described different methods (SEM, classical histology, cryomicroscopy, fluorescence, and confocal microscopy) for analyzing PLA-based CECs dynamically colonized by MSCs using a device developed by us [13]. The recombinant Tgfβ3 protein was used as a stimulator to induce chondrogenic differentiation of the cell culture, which is consistent with studies using the same cytokine to stimulate proliferation of chondrogenic cells [20,21].

The classical histology method with the preparation of paraffin blocks resulted in partial degradation of the polymer carrier. Although these preparations were relatively easy to produce, such a method seems to have significant limitations associated with the risk of partial degradation of the polymer carrier during sample preparation (as was demonstrated here). It may be possible to avoid degradation by improving the classical histology, but significant work is required to optimize the conditions (e.g., using reagents non-reactive with the polylactide membrane).

To prepare histological sections with another method in this study, we developed a new cryosectioning working protocol, different from the preparation of paraffin blocks, for analyzing both CECs and the joint fragments excised from experimental animals. The technique was optimized by including an additional polylysine layer, improving the adhesiveness to microscope slides and thus preventing tissue sections from peeling off the slides. Thus, the firm adhesion of the section to the slide was achieved. Cryosectioning methods were used according to the modified protocol to prepare CEC sections, allowing for their analysis without degradation during the preparation process. This protocol was thus shown to result in cryosections suitable for fluorescence microscopy analysis following both Alcian blue and hematoxylin and eosin staining. In particular, data from confocal microscopy were obtained, along with 3D video images of the CEC fragment with cells inside it. This technique made it possible to reveal the uneven colonization of the CEC by MSC culture and the dynamics of cell proliferation, which would have been impossible with classical histology due to the CEC’s degradation. Another advantage of this technique was the rapid production of the preparations. However, an additional adhesive layer applied onto the microscope slides was required. The resulting cryosections were stained with fluorescent dye and evaluated using fluorescent and confocal microscopy. The cryosection technique has advantages over standard histological processing, as it avoids the degradation of the polylactide carrier and, therefore, sample loss. According to recent studies from the literature, cryosections are increasingly being used to analyze cartilage structure and the level of chondrocyte proliferation. In particular, immunohistochemical staining of cryosections was used to study the influence of Tgfβ3 and FGF2 factors on the differentiation of chondrocytes in 3D hydrogels [22]. Cryosections of femoral heads were used in analyzing cartilage regeneration with immunohistochemical staining, as well as staining with Toluidine blue [23], safranin, and BCIP/NBT [24]. Comparison of the data obtained using conventional fluorescence microscopy and the results of confocal microscopy (after preparing a histological cryosection) revealed that the PLA carrier used exhibited extensive autofluorescence, which interfered with the analysis of the preparation. Thus, to obtain more accurate data on the signal level, it is preferable to use confocal microscopy to reliably separate the signal of the fluorescent label from the carrier autofluorescence (Figure 6).

SEM has been used for almost 50 years to study hyaline cartilage, with the first scientific article describing the structure of hyaline cartilage using this method published by Clarke in 1971 [25]. In our studies, SEM was shown to be highly effective in obtaining and demonstrating results without risking the degradation and loss of the preparation. A significant disadvantage of this method in analyzing CEC structures is that it is only able to be used to examine the surface (or near-surface) layer, due to the specificities of scanning microscopy. To obtain data on deeper layers, sample preparation is needed through a preliminary cut of the excess material, as shown by Clark and Simonian [26]. The advantage of this method is the simplicity and efficiency of producing a preparation. Using this technique, we were able to measure the defect size and estimate the characteristics of the naive carrier, which is consistent with literature data [27]. Reliable quantitative characteristics obtained for both the native scaffold and the CEC make it possible to theoretically calculate the mechanical properties of the prepared sample. This is important for the design of tissue-engineered constructs intended to replace hyaline cartilage defects and is consistent with the conclusions of other researchers [28]. We suggest that analysis of these data will help in finding a balance between the pore size, porosity, and mechanical characteristics of the obtained CEC, which is extremely important for the replacement of hyaline cartilage defects, as described by Ho and Hutmacher [28]. Another advantage of this technique is the ability to obtain illustrative data on the proliferation of a cell culture on the surface of a biodegradable carrier, which is consistent with the data from Cherubino et al. [29].

We also analyzed data from the in vivo experiments obtained using different methods after CEC transplantation into the area of simulated damage of the articular cartilage hyaline layer. The histological and SEM methods described have their own advantages and disadvantages. Thus, previously obtained data on the partial degradation of the polylactide carrier as a result of histological processing were confirmed. This is an additional limitation of said method in analyzing CEC structures based on polylactic acid. This phenomenon was more pronounced when analyzing CECs without their transplantation into the defect areas of experimental animals and less pronounced with prolonged cell proliferation on the joint surface after preliminary cell colonization. Optimal conditions as well as appropriate reagents that were not reactive with polylactide, could possibly enable the use of this method without significant limitations.

Another limitation of histological methods for the in vivo examination of tissue samples is their inability to accurately measure the geometric dimensions of the defect and regeneration areas and/or other perifocal changes. The HMs revealed different (lower) values for simulated defects compared to the SEM methods. Divergent results were obtained when trying to measure the quantitative characteristics of the simulated defects. We believe that the histological section was not strictly perpendicular to the center of the defect, which resulted in this discrepancy. Given the difficulties of ensuring that histological sections are exactly perpendicular to the center of small defects, we concluded that, under these restrictions, it is impossible to measure any size using histological data only, even in the presence of a scale bar. However, it should be noted that SEM does not allow for a precise assessment of the damage depth without preliminary sample preparation (a cross-section).

Moreover, it was difficult to obtain histological preparations appropriate for analysis of the knee-joint surface from rats with simulated defects that were 1 mm or smaller (such as the animals used in this study). In some cases (fewer than two out of six), the sections were produced above or below the damage, which led to the loss of the preparation’s relevance for further analysis. In contrast to classical histology, SEM made it possible to visualize and analyze the surface defect on the rat knee joint, since it was possible to independently view any part of the sample surface, and this was only limited by the size of preparation for the SEM studies (8 × 8 × 8 mm). Using a larger experimental animal, like a rabbit or sheep, would, of course, circumvent these problems, while significantly complicating and increasing the cost of the entire animal study.

The SEM method described is relevant and can be used along with classical histological methods, as shown in this study (Figure 7). The SEM method did not lead to carrier degradation, as was shown by the in vivo and in vitro results, and this was consistent with the data obtained by Ho et al. [28]. The results obtained by this group were the same when assessed on a special ICRS scale and on the O’Driscoll scale. We suggest that this was due to the avascular structure of hyaline cartilage and its connection with the internal structure of the regenerating tissue in the damaged area, along with its ability to withstand significant mechanical stress for a long time. This indicates that SEM can be used as an independent method for assessing the regenerative processes on the surface of simulated damage and/or a CEC construction transplanted to hyaline cartilage. However, the SEM method does not make it possible to discern specific morphological characteristics of the regenerating tissue, nor its cellular composition, extracellular matrix synthesis, the formation of underlying bone, the subchondral lamina, etc. Nevertheless, this problem seems to be less important in the context of the screening or primary analysis of the experimental application of CECs. The CEC effectiveness/efficiency (for the replacement of avascular hyaline tissue) is the main result, and this can be assessed by researchers in accordance with the available scale, where the score of such an assessment (ICRS) will correlate with the results obtained by classical histological methods (O’Driscoll). When analyzing regenerative changes in vivo, SEM provides the precise surface structure. This technique is reliable and makes it possible to accurately determine quantitative characteristics of the area of damage (or regeneration); for example, size, diameter, and depth (in some cases). Unfortunately, it is impossible to analyze the processes occurring under the outer layer of the extracellular matrix and/or cell culture without special techniques. However, processes taking place in the hyaline cartilage (where there are not many cells and a large amount of extracellular matrix) show that its surface structure directly depends on the internal structure and proliferation of the tissue, since it is under constant and significant mechanical stress.

With chondrogenic cell proliferation inside the CEC (implanted in an animal in vivo), this implant will be resistant to external forces and will not degrade under significant mechanical stress; therefore, in SEM images, a reduction in the size of the defect will be seen. In cases of incorrect (ineffective) proliferation of the CEC (implanted in an animal in vivo), this implant will be susceptible to external loads and will degrade under significant mechanical loads. In this case, the SEM image will show significant degenerative damage to the surface layer.

This assumption is valid only for those tissues that simultaneously satisfy the following conditions: (1) they contain few cells and a large amount of extracellular matrix; (2) the observation period is “medium-term” or longer; and (3) the tissue is exposed to significant mechanical stress. However, for hyaline cartilage, these conditions are exactly fulfilled, which, in our opinion, significantly simplifies the task by making it possible to analyze SEM images of regenerative changes in the damaged area during CEC implantation.

This indicates that the SEM method can be used to analyze regenerative processes following CEC transplantation into the area of a hyaline cartilage defect. We suggest that this technique can be used independently of histological data; however, this hypothesis needs to be further confirmed by analyzing more data for different periods and different groups. Nevertheless, to obtain a more complete picture, it would be beneficial to conduct various parallel studies and compare their results, as described by Goodwin and Ho [28,30].

## 5. Conclusions

Experimental approaches to restoring hyaline cartilage using CECs based on a biodegradable PLA polymer and cell culture are currently being extensively studied. Histological analysis is one of the classical methods for evaluating the regenerative changes and the effectiveness of the application of CECs to areas of simulated damage. However, classical histological processing leads to partial degradation of the polymer carrier; therefore, the preparation and assessment of cryosections using fluorescence and confocal microscopy seems to be more suitable for effective data analysis. PLA hydrolyzes in the presence of many organic solvents (including xylene and 50% ethanol/water mix), which makes the polymer matrix swell, increasing chain mobility and rapid solvent-induced crystallization [31,32]. SEM is another method for assessing the regeneration of hyaline cartilage. We demonstrated the correlation between the data obtained using classical histological methods and using scanning electron microscopy. Our comparative analysis of the different methods used to analyze cell-engineered constructs will be useful for an adequate assessment of their effectiveness in the restoration of hyaline cartilage.

## Data Availability

The data that support the findings of this study are available from the corresponding author, M.S. Bozhokin, upon reasonable request.

## Supplementary Materials

The following are available online at https://www.mdpi.com/article/10.3390/mps4040077/s1. Figure S1. The picture of polyethylene terephthalate mold used for scaffold preparation, Figure S2. Schematic diagram of the cell engineered construct preparation (a) and the inner view of the device (b), Figure S3. SEM photo of the defect area on the 90th day of observation after the creation of a model defect with a boron with a diameter of 1.0 mm. Degenerative changes on the surface of the condyle of the joint are visible, associated with further destruction of the articular surface of the hyaline cartilage. Part of the condyle of the joint is missing. Damaged area shown in circle has the diameter over 3.0 mm, Figure S4. SEM photo of the defect area on the 90th day of observation after the CEC transplantation. A rounded area with a diameter of 1.2 mm is visualized on the surface of the condyle. The surrounding cartilage is not visually damaged, there are no obvious degenerative changes, in the area of the damage, marginal and circumferential cracks along the perimeter of the damage are noted.
