# Supplementary material for: Specificities of Scanning Electron Microscopy and Histological Methods in Assessing Cell-Engineered Construct Effectiveness for the Recovery of Hyaline Cartilage
Source: Methods Protoc. 2021 Oct 27;4(4):77. doi: 10.3390/mps4040077 (PMC8628887; doi:10.3390/mps4040077)
Supplement: Supplementary file 1 [file mps-04-00077-s001.zip › mps-1372088-SI.pdf]

Supplementary Materials

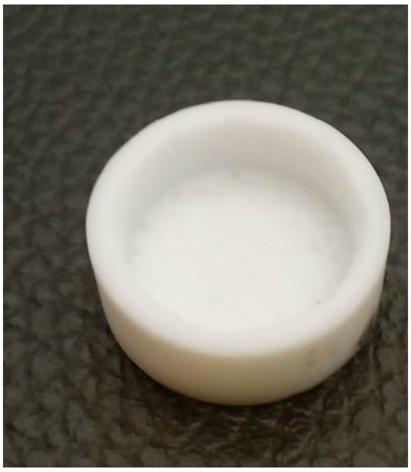

Figure S1. The picture of polyethylene terephthalate mold used for scaffold preparation.

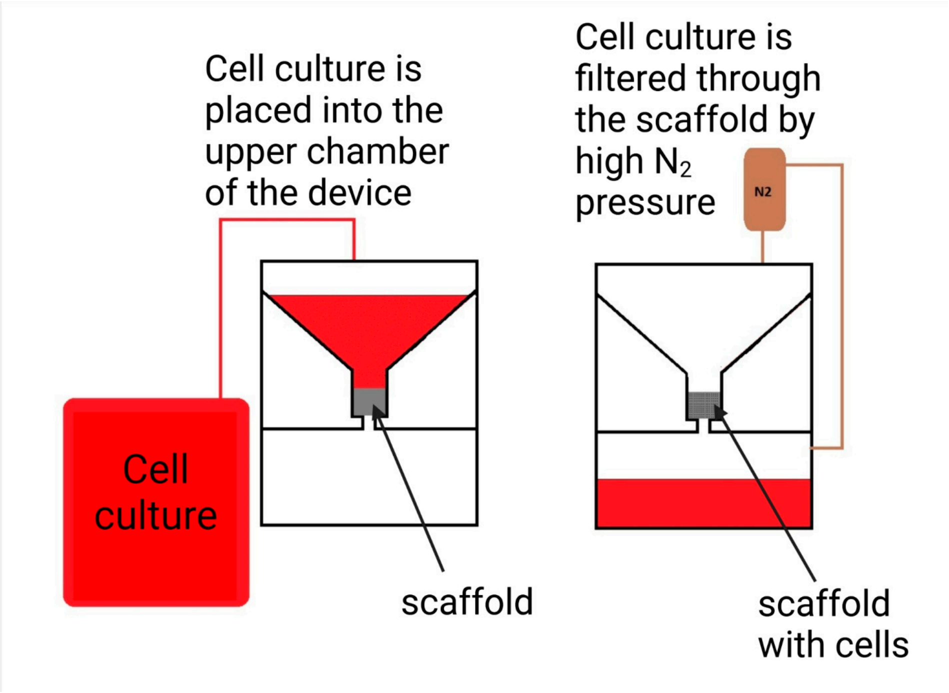

(a)

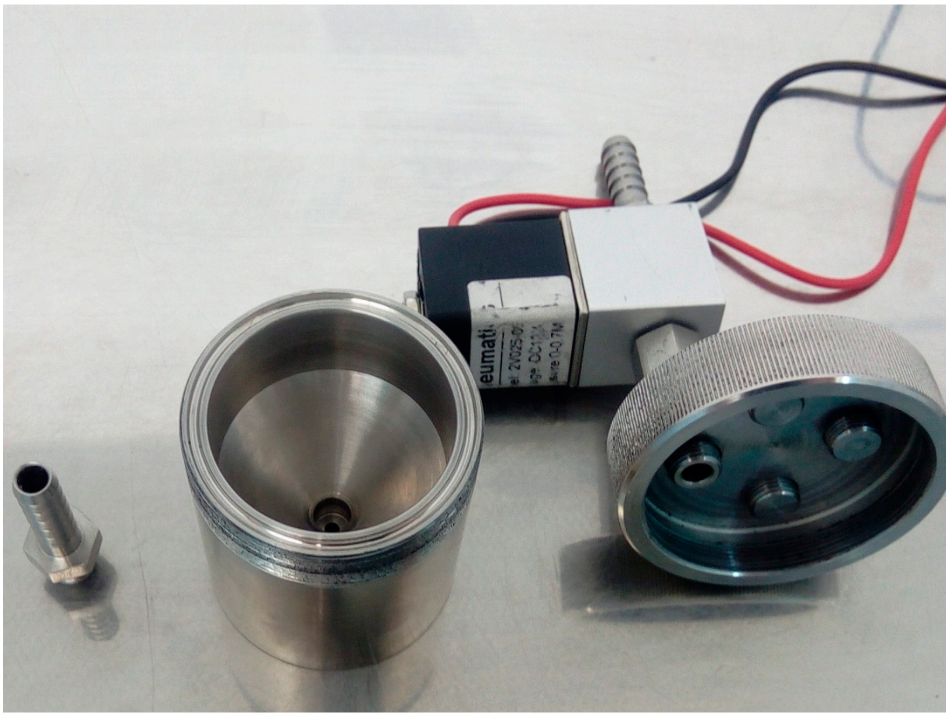

(**b**)

Figure S2. Schematic diagram of the cell engineered construct preparation (**a**) and the inner view of the device (**b**).

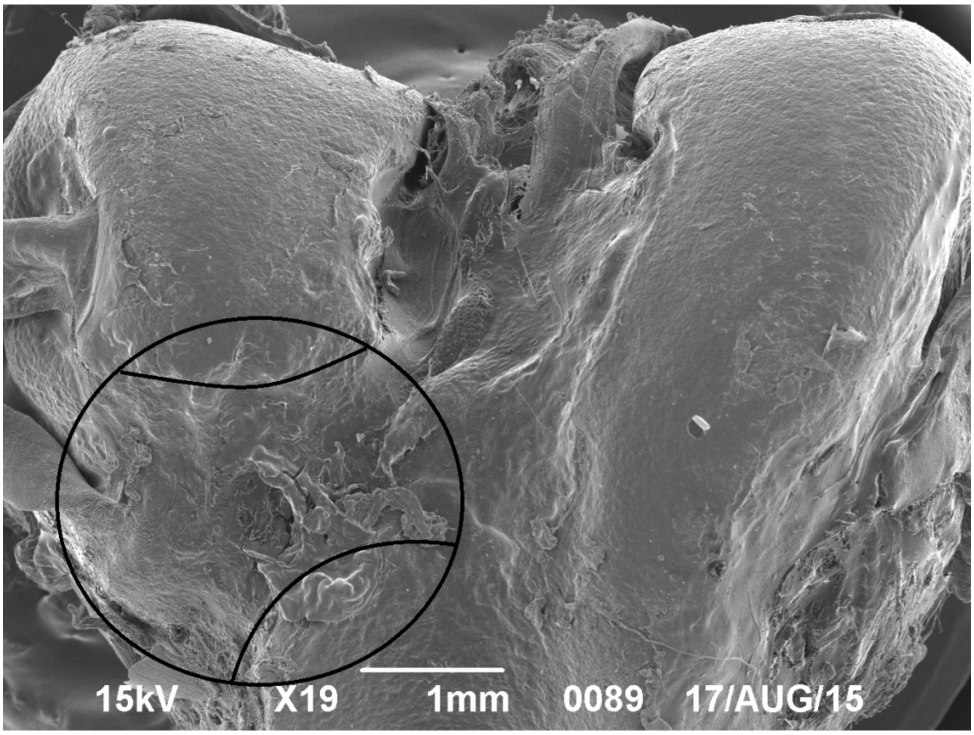

Figure S3. SEM photo of the defect area on the 90th day of observation after the creation of a model defect with a boron with a diameter of 1.0 mm. Degenerative changes on the surface of the condyle of the joint are visible, associated with further destruction of the articular surface of the hyaline cartilage. Part of the condyle of the joint is missing. Damaged area shown in circle has the diameter over 3.0 mm.

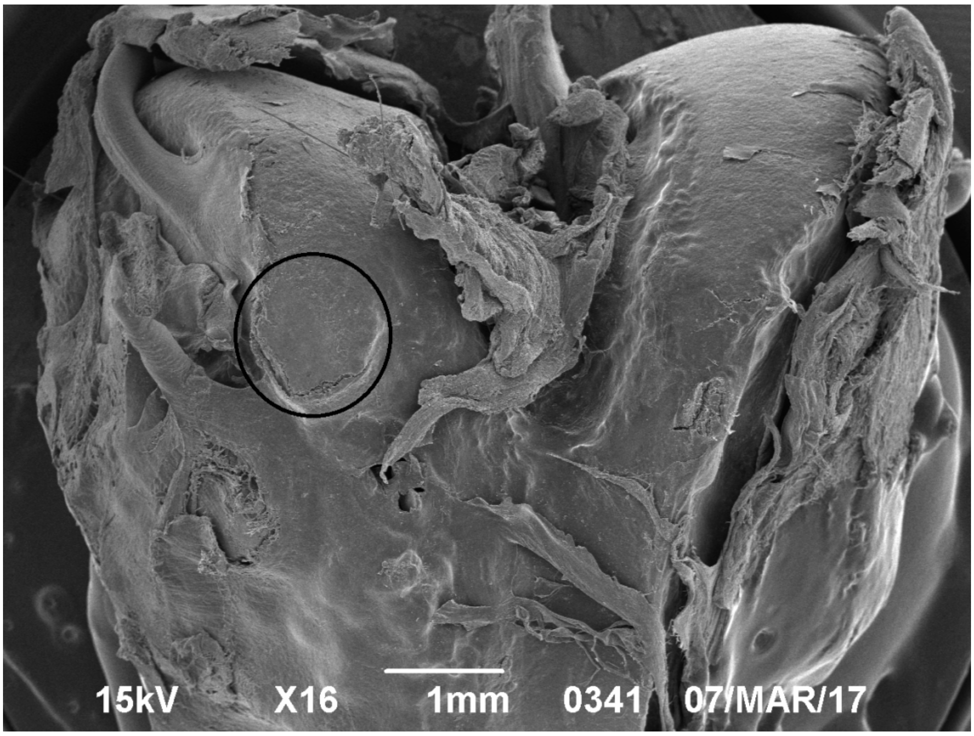

Figure S4. SEM photo of the defect area on the 90th day of observation after the CEC transplantation. A rounded area with a diameter of 1.2 mm is visualized on the surface of the condyle. The surrounding cartilage is not visually damaged, there are no obvious degenerative changes, in the area of the damage, marginal and circumferential cracks along the perimeter of the damage are noted.
